# Supplementary figures and images for: Evolution of Functional Diversity in the Holozoan Tyrosine Kinome
Source: Mol Biol Evol. 2021 Sep 13;38(12):5625–39. doi: 10.1093/molbev/msab272 (PMC8662651; doi:10.1093/molbev/msab272)

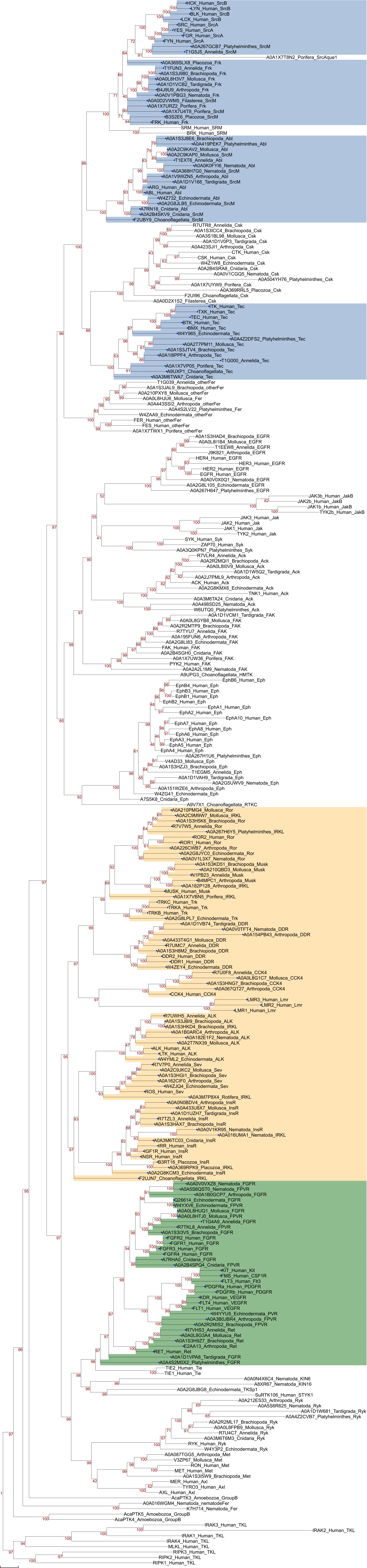

Supplement: msab272_Supplementary_Data [file msab272_supplementary_data.zip › supp file s4 - holozoan tk tree.pdf]
